# Impact of external odor on self‐grooming of lesser flat‐headed bats, *Tylonycteris pachypus*

**DOI:** 10.1002/ece3.5377

**Published:** 2019-06-25

**Authors:** Jie Liang, Jian Yang, Huanwang Xie, Xingwen Peng, Xiangyang He, Yunxiao Sun, Libiao Zhang

**Affiliations:** ^1^ Guangdong Key Laboratory of Animal Conservation and Resource Utilization, Guangdong Public Laboratory of Wild Animal Conservation and Utilization Guangdong Institute of Applied Biological Resources Guangzhou China

**Keywords:** bat, odor, scent mark, self‐grooming

## Abstract

Grooming is a common behavior of some mammals. Previous studies have shown that grooming is a means by which animals clean themselves, remove ectoparasites, and lower their body temperature. It is also involved in olfactory communication. Bats belong to the order Chiroptera and, like most mammals, are the natural host of many ectoparasites. Bat grooming, including licking and scratching, is one of the ways to reduce the adverse effects caused by ectoparasites. Bat grooming may also be induced by exogenous odor. In this study, we used lesser flat‐headed bats (*Tylonycteris pachypus*) to test the hypothesis that exogenous odor affects the self‐grooming behavior of bats. Results showed that external odor from distantly related species caused lesser flat‐headed bats to spend more time in self‐grooming. Lesser flat‐headed bats that received odor from humans spent the longest time in self‐grooming, followed by those that received odor from a different species of bats (*T. robustula*). Lesser flat‐headed bats that received odor form the same species of bats, either from the same or a different colony, spent the least amount of time in self‐grooming. These results suggest that bats can recognize conspecific and heterospecific through body scent.

## 1 INTRODUCTION

Grooming is a behavior in which animals clean themselves or their peers by licking and scratching their fur and skin (Kalueff et al., 2016; Yu, Yue, Sun, & Zhao, 2010; Zhang, Zhang, Tang, & Hong, 2013). This behavior can be allogrooming or self‐grooming (Scheider, Waller, Ona, Burrows, & Liebal, 2016). In allogrooming, animals of the same species groom each other. Allogrooming plays an important role in maintaining social relationships (Ventura, Majolo, Schino, & Hardie, 2005). Self‐grooming of animals may occur anytime, but most frequently (Ferkin, 2006; Yu et al., 2010) occurs after feeding or while exploring their surroundings. It can be evoked when animals encounter exotica or feel stressed (Kalueff et al., 2016; Radford, 2012; Song, Berridge, & Kalueff, 2016). Self‐grooming in rodents may be triggered by meeting conspecifics or encountering scent from their peers (Carter et al., 1989; Ferkin, 2006; Ferkin, Leonard, & Gilless, 2007; Steiner, 1973). Previous studies have shown that self‐grooming is a mechanism by which bats remove ectozoa (Czenze & Broders, 2011; Yu et al., 2010; Zhang et al., 2013). Most mammals discriminate their kin by olfactory cues (Busquet & Baudoin, 2005; Hurst & Beynon, 2010). Kin recognition facilitates cooperation and avoids competition or inbreeding among relatives (Gerlach & Lysiak, 2006; Isles et al., 2002; Leclaire, Nielsen, Thavarajah, Manser, & Clutton‐Brock, 2013; Olsén, Grahn, Lohm, & Langefors, 1998). The relationship between olfactory‐based kin recognition and self‐grooming has not been well studied.

In this study, we investigated the effect of exogenous odor on self‐grooming and the correlation between self‐grooming and olfactory recognition in lesser flat‐headed bats (*Tylonycteris pachypus*, Vespertilionidae, Chiroptera). Most species of bats are gregarious and live in colonies. Lesser flat‐headed bats form long‐term harem groups in hollow bamboo internodes (Medway & Marshall, 1972; Zhang, Liang, Zhou, Lu, & Zhang, 2004) with 2–24 bats in each colony. In the limited space of bamboo internodes, bats need to recognize their mates and to prevent others that are not conspecifics from entering their roost. Lesser flat‐headed bats never roost with sympatric siblings such as greater flat‐headed bats (*T. robustula*; Medway & Marshall, 1970; Zhang et al., 2004). This phenomenon indicates that bats have species recognition mechanisms. Previous studies showed that there are not many differences among the colonies of lesser flat‐headed bats (Medway & Marshall, 1970, 1972; Zhang et al., 2004). However, we found that the sex ratio at birth was near equal, while more females in adult during our surveys. Thus, we also look for differences among all of the colonies, including sex ratio and size.

We hypothesized that lesser flat‐headed bats discriminate conspecific and heterospecific through their external odor and perform different degrees of odor‐covering by self‐grooming. To test this hypothesis, we determined the effects of exogenous odor on the amount of time that lesser flat‐headed bats engage in self‐grooming. We also investigated whether self‐grooming of lesser flat‐headed bats is a mechanism of their species recognition.

## 2 METHODS

### 2.1 Bat collection

**Figure 1:**
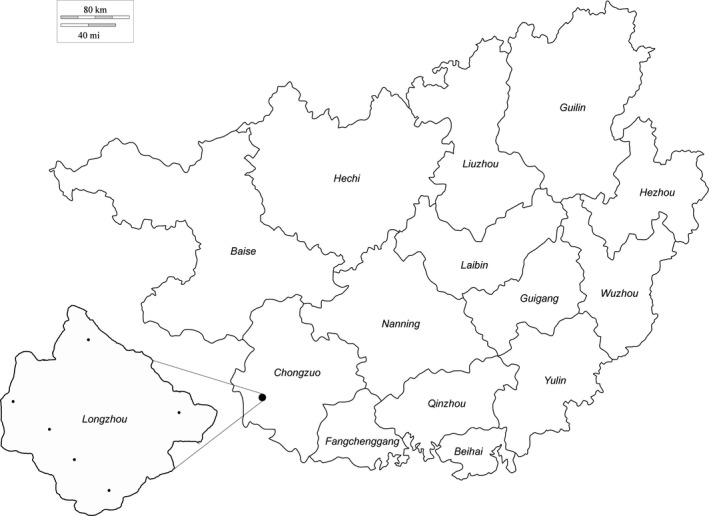
Bat collection sites. Dots indicate six bat sampling sites in Longzhou County, Guangxi Province, China

Bats were collected from September to October 2011 from Longzhou County, Guangxi Province, China (Figure 1), where heavily cultivated bamboos provide roosts to lesser flat‐headed bats.

Greater flat‐headed bats, *T. robustula*, belong to the same genus as *T. pachypus* and usually share the same bamboo forest in Longzhou County with lesser flat‐headed bats. They were collected to serve as external odor suppliers. Forearm length and body weight of each bat were measured (to 0.1 mm and 0.1 g, respectively; Zhang et al., 2013).

### 2.2 Experimentation

Because odor from different genders may affect results, external odor was collected only from male bats. To collect odor, the muzzle, external genitalia, and anus of a bat were each scrubbed with a cotton swab for 15 times. In the same manner, odor from humans was collected from the head, nose, and axilla of a person. Each external odor swab was saved in a clean beaker.

Each bat was used for only one odor collection, and the same bat was not used for grooming experiment. For humans, odor was collected only once a day from a person.

**Figure 2:**
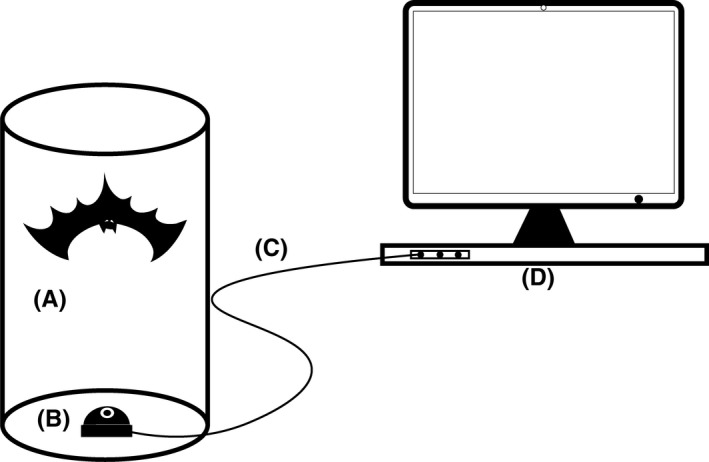
Monitoring equipment for observation of self‐grooming. A: bamboo tube, B: infrared camera, C: data link, and D: computer monitor

Bats were divided into five groups (42–55 bats per group): (a) control group (CG), (b) conspecifics from different colonies in the same forest roost (DCG), (c) conspecifics from different roosts (DRG), (d) *T. robustula* (TRG), and (e) human group (HG). To apply odor to a bat, the bat was scrubbed 30 times each on dorsum and belly with one external odor swab. Each CG bat was scrubbed with a clean cotton swab without odor. Each DCG bat was stimulated with odor from *T. pachypus* from different colonies in the same forest roost, while each DRG bat was stimulated with odor from *T. pachypus* of different roosts. TRG bats were stimulated with *T. robustula* odor, and HG bats were stimulated with human odor. Each lesser flat‐headed bat (*T. pachypus*) that received odor stimulation was placed in apparatus A (Figure 2). The grooming behavior of the bat in apparatus A was observed through the monitor for 15 min. When a test was completed, apparatus A and all other materials were washed with clean water, rinsed with 75% alcohol, and air‐dried before they were reused.

### 2.3 Statistical analysis

The Kolmogorov–Smirnov (KS) test was used to determine whether two data sets differ significantly. The difference in forearm length and body weight of bats among different groups was analyzed by one‐way ANOVA and between groups by Tukey's test. The *t* test was used to determine whether there was a significant difference in the length of self‐grooming time between male and female bats. All values are presented as mean ± *SE*, and *α* < 0.05 was the lowest acceptable significance level.

## 3 RESULTS

In total, 244 lesser flat‐headed bats were used in this study. Among them, 177 were females, and 67 were males. There was no significant difference in forearm length and body weight among the five groups or between groups (ANOVA: forearm length, *F* = 2.150, *p* > 0.05; body weight, *F* = 1.850, *p* > 0.05; Tukey: all *p* > 0.05; Table 1).

**Table 1:** Morphological comparison among groups (mean ± *SE*)

Group (*n*)	Forearm length (mm)	Body weight (g)
CG (55: 18♂, 37♀)	26.43 ± 0.06	3.74 ± 0.03
DCG (49: 18♂, 31♀)	26.51 ± 0.08	3.67 ± 0.04
DRG (48: 8♂, 38♀)	26.44 ± 0.07	3.80 ± 0.04
TRG (42: 12♂, 30♀)	26.29 ± 0.08	3.76 ± 0.04
HG (52: 11♂, 41♀)	26.28 ± 0.07	3.78 ± 0.03
*F*	2.150 ns	1.850 ns

Abbreviation: ns: not significant.

There was no significant difference in self‐grooming time between male and female bats in CG, DCG, DRG, and TRG groups (*t* test: all *p* > 0.05). Only the HG bats showed a significant gender difference in self‐grooming time, with males spending more time in self‐grooming (males: 434.55 ± 15.73, females: 383.32 ± 9.09, *t* test: *t* = 2.644, *p* < 0.05; Table 2).

**Table 2:** Self‐grooming time comparison between male and female bats within group (mean ± *SE*)

Group	Self‐grooming time (male/female)^a^	*t* value
CG (55: 18♂, 37♀)	117.44 ± 24.15/94.73 ± 72.59	0.948 ns
DCG (49: 18♂, 31♀)	217.44 ± 9.72/215.52 ± 9.31	0.135 ns
DRG (48: 8♂, 38♀)	254.63 ± 31.18/215.05 ± 13.66	1.99 ns
TRG (42: 12♂, 30♀)	357.25 ± 26.07/305.23 ± 13.74	1.912 ns
HG (52: 11♂, 41♀)	434.55 ± 15.73/383.32 ± 9.09	2.644*

Abbreviation: ns, not significant.

a Self‐grooming time in seconds within 15 min of observation.

* *p* < 0.05.

**Figure 3:**
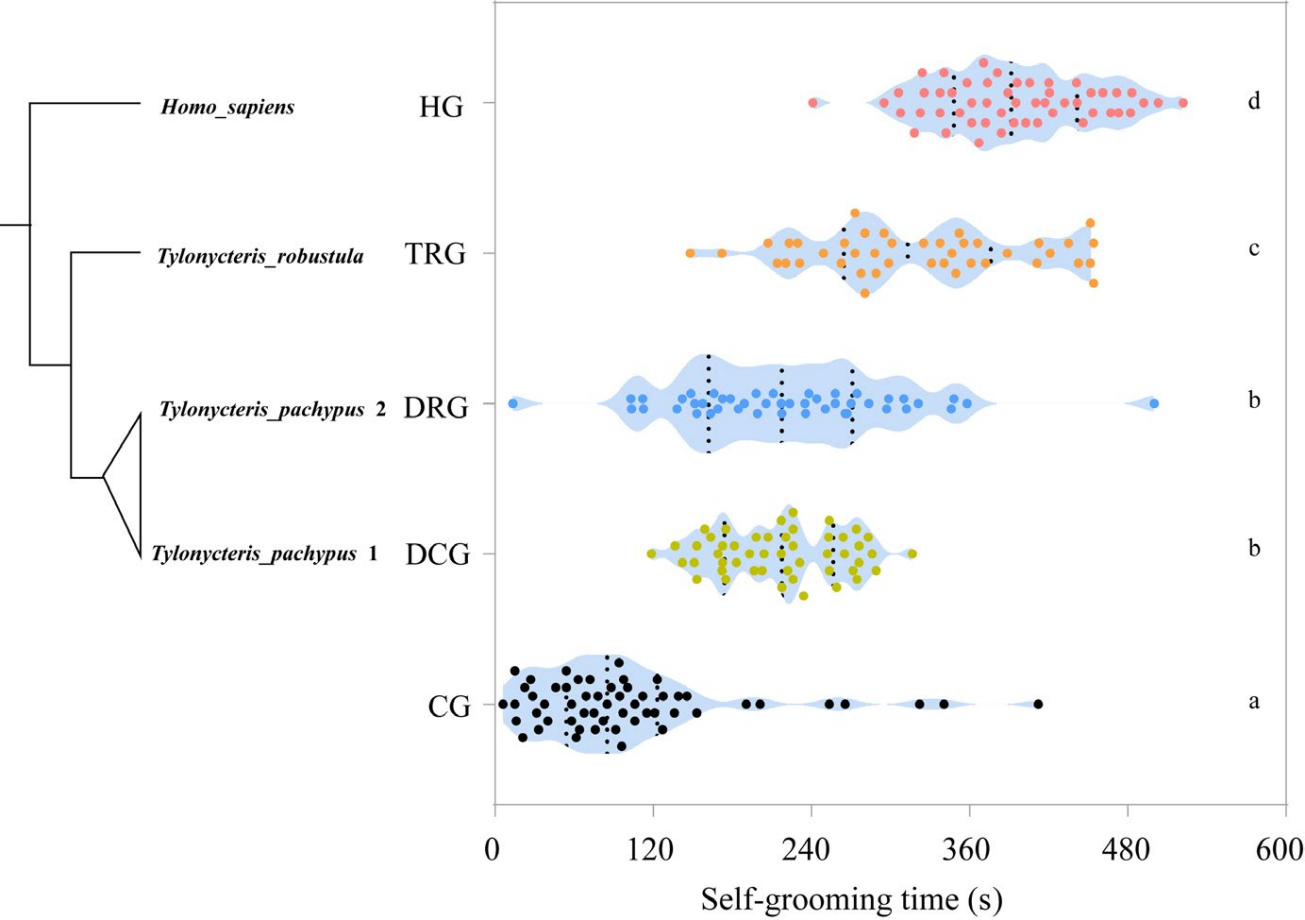
Difference in self‐grooming time among groups (mean ± *SE*). Different letters (a, b, c, and d) indicate significant difference, and the same letter indicates no significant difference

There was a significant difference in the length of self‐grooming time among the five groups of bats (ANOVA: *F* = 121.010, *p* < 0.05; Figure 3). Human group bats spent the longest time in self‐grooming (394.15 ± 8.37 s), while CG bats spent the shortest (102.16 ± 11.22 s). The amount of self‐grooming time was not significantly different between DCG (216.22 ± 6.82 s) and DRG bats (221.93 ± 12.57 s; Tukey: *p* > 0.05). The average self‐grooming time of each of these two groups (DCG and DRG) was longer than that of CG bats (Tukey: both *p* < 0.05) and shorter than that of TRG bats (320.10 ± 12.68 s, Tukey: both *p* < 0.05).

## 4 DISCUSSION

In this study, we found that females were the majority in colonies of lesser flat‐headed bats. This may be due to the harem mating system of bats (Medway & Marshall, 1972). There was no significant difference in morphology among the lesser flat‐headed bats used in this study. The amount of time that lesser flat‐headed bats spent in self‐grooming was significantly different when they were exposed to different kinds of external odors. The bats in the control group (CG) that were not exposed to any external odor spent the least amount of time in self‐grooming. The amount of time that lesser flat‐headed bats spent in self‐grooming when they were exposed to odor from conspecifics of the same colony or a different colony was similar. When lesser flat‐headed bats were exposed to the odor from different species of organisms, that is, *T. robustula* bats or humans, they spent more time in self‐grooming. Generally, the odor from organisms of more distantly related kinship caused a longer self‐grooming time. This observation suggests that external odor does affect self‐grooming of lesser flat‐headed bats and that bats can distinguish odors from different organisms. During self‐grooming, lesser flat‐headed bats licked their fur and scratched their patagium with their tongue intensely, presumably to replace the artificially applied exogenous odor with their own odor to avoid peer rejection.

Sexual difference in olfactory capacity of most bats used in this study was not significant. When exposed to odor from conspecific or heterospecific males, only the lesser flat‐headed bats in the HG group showed a significant gender difference in self‐grooming, with males spending more time (434.55 ± 15.73 vs. 383.32 ± 9.09). Female and male bats in the other four groups responded similarly to odors from males of conspecifics or sibling.

Previous studies showed that the frequency of self‐grooming in rodents, such as ground squirrels and hedgehogs, is higher when they encounter odor from opposite‐sex conspecifics than when they encounter odor from same‐sex conspecifics (Brockie, 1976; Ferkin, 2006; Ferkin, Sorokin, & Johnston, 1996; Steiner, 1974; Yu et al., 2010). This is may be due to the possibility that self‐grooming is a means by which animals communicate with opposite sex and select mates (Achiraman et al., 2014; Ferkin, 2006, 2018; Yu et al., 2010). Therefore, in general, animals respond more intensely to the odor from opposite sex (Ferkin, 1992). However, our study revealed that male and female lesser flat‐headed bats spent similar amount of time in self‐grooming when they were exposed to male odor of conspecifics or *T. robustula*, while male lesser flat‐headed bats spent more time than females in self‐grooming when they were exposed to male odor of humans.

Discrimination between relatives and nonrelatives is crucial for animal social interaction; it facilitates cooperation among relatives and helps animals recognize their kin and avoid inbreeding (Hamilton, 1964; Waldman, 1988). Familiarity and phenotype matching are the most common mechanisms for animal kin recognition (Leclaire et al., 2013). A previous study demonstrated that the major histocompatibility complex (MHC) plays a role not only in immunological responses but also in mate choice and odor‐based kin recognition in several animal species (Gerlach & Lysiak, 2006; Milinski, Griffiths, Reusch, & Boehm, 2010), such as sticklebacks. Some animals, such as mice (*Mus musculus*; Yamazaki et al., 1979) and humans (Wedekind, Seebeck, Bettens, & Paepke, 1995), have been shown to differentiate conspecific odors differently due to disparate MHC compositions. A study showed that mice prefer mating partners with the same major urinary protein (MUP), which is a species‐specific kinship marker (Green et al., 2015). Our study demonstrated that bats can detect differences among various external odors, suggesting that bats may recognize species through odor, MUP, or other kinship markers. Mechanisms for such discrimination remain to be investigated.

In conclusion, we demonstrated that exogenous odors can affect the self‐grooming behavior of lesser flat‐headed bats. Exogenous odors from distantly related species, such as a different species of bats or humans, led to a longer self‐grooming time. Our results also suggest that lesser flat‐headed bats can recognize their relatives through odor. Results of this study provide new directions for research on odor‐based communication and species recognition in bats.

## CONFLICT OF INTEREST

None declared.

## AUTHOR CONTRIBUTIONS

Libiao Zhang and Jian Yang designed this study. Jian Yang and Xiangyang He performed the experiments. Jie Liang, Xingwen Peng, and Yunxiao Sun analyzed the data, Libiao Zhang, Jie Liang and Huanwang Xie wrote the manuscript.

## ETHICAL APPROVAL

Collection of bats was done according to the guidelines of Regulations for the Administration of Laboratory Animals (Decree No. 2, State Science and Technology Commission, People's Republic of China). The animal experiments performed in this study were approved by the Guangdong Entomological Institute Administrative Panel on Laboratory Animal Care (No. GDEI‐AE‐2006001).

## Data Availability

Data from this study have been uploaded in Dryad Digital Repository (https://doi.org/10.5061/dryad.67r78cn).
